# Progress toward realizing the promise of decentralized clinical trials

**DOI:** 10.1017/cts.2023.706

**Published:** 2024-01-14

**Authors:** Matthew W. McCarthy, Christopher J. Lindsell, Radha Rajasingham, Thomas G. Stewart, David R. Boulware, Susanna Naggie

**Affiliations:** 1 Department of Medicine, Weill Cornell Medicine, New York, NY, USA; 2 Duke Clinical Research Institute, Durham, NC, USA; 3 Department of Biostatistics and Bioinformatics, Duke University, Durham, NC, USA; 4 Division of Infectious Disease & International Medicine, Department of Medicine, University of Minnesota, Minneapolis, MN, USA; 5 University of Virginia School of Data Science, Charlottesville, VA, USA; 6 Department of Medicine, Duke University, Durham, NC, USA

## Introduction

Over the last decade, decentralized clinical trials have risen in popularity. Sometimes referred to as virtual, siteless, or direct-to-participant trials, decentralized trials are characterized by features that bring trial activities to the participant rather than bringing the participant to study sites. The promise is that every person has access to clinical trials, and that clinical trials are able to enroll a diverse and representative population. Whether or not this promise is being realized has yet to be established. The science guiding the design and deployment of decentralized trials is still in its infancy, although experience has accelerated due to the severe acute respiratory syndrome coronavirus 2 (SARS-CoV-2) pandemic.

The arrival of SARS-CoV-2 left in its immediate wake a plethora of abandoned research studies. Those trials that could continue accrued slowly, and investigators had to adapt methods and processes to no- or low-touch approaches. Little experience existed on how to adapt to a decentralized approach, although serendipitously the FDA had issued guidance for electronic Informed Consent (eConsent) in 2016, and the Clincial Trials Transformation Iniative published their recommendations for decentralized trials in 2018 [1,2].

With the science of decentralized trials only just beginning to emerge, the lack of experience conducting trials without bringing patients to trial sites meant that in the early months of the pandemic, there was a dearth of robust, rigorous, generalizable studies to address fundamental questions about coronavirus disease 2019 (COVID-19) treatment, transmission, and epidemiology. The medical community was reliant on smaller, poorly designed studies to provide urgent answers to questions about treatments that could save lives and keep people out of hospitals. The need for well-designed trials was not in balance with the on-the-ground reality of limited infrastructure, limited supplies, limited personnel, and no access to healthcare facilities for non-urgent activities, including research.

As COVID-19 was sweeping the world, the scientific community, in partnership with sponsors, industry, academicians, patients, and scores of other stakeholders, united to launch studies that would translate into meaningful advances for a novel disease. Lockdowns, overextended healthcare systems, an exhausted workforce, and uncertain viral transmission dynamics meant that clinical research could not be conducted as usual. Given these substantial infrastructure and personnel constraints, investigators turned to decentralized clinical trials, where some or all research activities occur at nontraditional sites, including in the homes of participants. The unmet need for decentralized approaches created by the pandemic, coupled with the rapid normalization of telemedicine and advances in technology for data collection, storage, and transmission ushered in a dynamic era during which decentralized approaches to clinical trial operations and oversight have now been extensively deployed.

This thematic issue of the *Journal of Clincial and Translational Science* includes a collection of articles exploring the many innovations, lessons learned, and implications of the rapid escalation of decentralized trial activities. The collection provides novel insights into decentralized clinical trial design and oversight, recruitment and retention, participant engagement and informed consent, study activity operations including drug delivery, patient-reported outcomes, adverse event reporting, and many other issues.

## Diversity, representativeness, and access

One major goal of decentralized trials, regardless of the pandemic context, is to increase the diversity and representativeness of participants, including through expanded accessibility and increased geographic reach. In their systematic literature review, Miyata, Tafuto, and Jose identified 12 studies reporting on the the influence of decentralized approaches on recruitment, retention, diversity, and reach [3]. Experience consistently shows that decentralized approaches improved all domains in the identified studies. Fahey *et al.* delved into recruitment methods for decentralized trials and argued that they may not always achieve the sociodemographic reach expected, but that a small unconditional monetary gift provided at the time of recruitment can increase recruitment efficiency at low overall cost [4]. Hanley *et al.,* reporting the experience of National Center for Advancing Translational Sciences (NCATS)’ Trial Innovation Network, also suggest caution in assuming that decentralized trials have solved the problems of diversity in recruitment and retention – at least in their current incarnation – calling for more research on this topic [5]. To understand barriers to successful diverse and representative recruitment, it is critical to hear from potential participants in relevant communities. Hamm *et al.* describe stakeholder engagement activities conducted for a decentralized trial, with the work done in a decentralized manner [6].

While Hanley *et al.* and ACTIV-6 describe the potential for geographic diversity and reach, it is recognized that scaling geographic reach may be limited by differences in state-level policies and practices. Zawada *et al*., report a framework to address this barrier, although it is clear much work is needed to overcome the “nonuniform policy landscapes that modulate the scope and scale of DCTs on a state-by-state basis in the United States” [7].

## Innovations and deployment at scale

A number of decentralized trial innovations and deployments are also described in this issue. For example, Dulko *et al.* describe the creation of a toolset that can be used to deploy a decentralized trial, which they refer to as a “clinical-trial-in-a-box” [8]. Similarly, Cafaro *et al.* describe their approach to conducting a single center trial in a decentralized manner, and through their report are making public the tools they developed to support trial activities [9]. Two large, decentralized trials, COVID-OUT and ACTIV-6, have provided critical evidence about the effectiveness of repurposed medications in keeping patients with SARS-CoV-2 infection out of the hospital and helping them feel better faster [10]. Both demonstrate the capacity for decentralized trials to reach every state of the US, and to be deployed internationally. Of course, decentralized methods are not restricted to testing treatments. Soni *et al.* describe how they were able to enroll a large population using a digital, siteless approach to generate regulatory-quality data on the performance of rapid antigen tests for SARS-CoV-2 [11].

Leveraging the acceptability of telemedicine for clinical care, a literature review by Cummins *et al.* notes the power of this technology for clinical research and summarizes its many uses [12]. In another study reported in this issue, Cummins *et al.* describe the breadth of technologies now being incorporated into clinical research to support decentralization, including centralized regulatory management, electronic signatures and consent, online payment systems, electronically delivered patient-reported outcome measures, wearables, and even home health nursing portals [13]. As innovations needed to bring the research to participants rather than the other way around continue to evolve, it will be important to evaluate whether they are achieving the intended goal of diverse, representative trial participants and trials available to all.

## Realizing the promise

The expectation is that decentralized trials are less resource-intensive and expand access to clinical trials by lowering barriers to engagement, recruitment, and retention. This is made possible by decreasing complexity, limiting or eliminating the need for participants to travel to sites, and limiting high-touch study interactions. The expanded access to clinical trials is expected to benefit the general population, increasing access to therapies that may not otherwise be available. In lowering barriers to study participation, opportunities for research engagement should be available to a wider community, increasing the diversity of the study population. Study outcomes that can be reliably collected in a remote trial and accepted by regulatory authorities are emerging, supported by newly available draft guidance from the FDA [14].

The types of interventions that are appropriate for decentralized clinical trials still require appropriate consideration to ensure safety of participants, and workflows and protocols for ensuring adequate safety reporting is critical. While the benefits of decentralized trials are many, challenges remain common and the current science of decentralized trials suggests there is some way to go before research studies are potentially available to every person in the US regardless of community or geography. We are excited that the landscape for clinical trial innovation is not just supportive of bringing research to people, but that agencies such as ARPA-H are leading major initiatives to continue growing the nation’s decentralized clinical trial ecosystem in a fair and equitable manner. To ensure the promises of diversity, representativeness, and geographic reach are met, we encourage the ongoing evaluation and publication of methods used to support decentralized clinical research.

## References

[1] United States Food and Drug Administration. Use of Electronic Informed Consent in Clinical Investigations - Questions and Answers. (https://www.fda.gov/regulatory-information/search-fda-guidance-documents/use-electronic-informed-consent-clinical-investigations-questions-and-answers). Accessed November 30, 2023.

[2] Clinical Trials Transformation Initiative. CTTI Recommendations: Dencentralized Clinical Trials. CTTI Recommendations: Decentralized Clinical Trials. (ctti-clinicaltrials.org). Accessed November 30, 2023.

[3] Miyata BL , Tafuto B , Jose N. Methods and perceptions of success for patient recruitment in decentralized clinical studies. J Clin Transl Sci. 2023;7(1):e232,1–9. doi: 10.1017/cts.2023.643. PMC10643920 38028356

[4] Fahey MC , Dahne J , Chen BK , Smith TT , Wahlquist AE , Carpenter MJ. And then I found $5’: optimizing recruitment efficiency in remote clinical trials. J Clin Transl Sci. 2023;7(1):e102. 37250999 10.1017/cts.2023.533 PMC10225265

[5] Hanley DF , Bernard GR , Wilkins CH , et al. Decentralized clinical trials in the trial innovation network: value, strategies, and lessons learned. J Clin Transl Sci. 2023;7(1):e170. 37654775 10.1017/cts.2023.597 PMC10465321

[6] Hamm ME , Arnold J , Denson J , et al. The ACTIV-6 stakeholder advisory committee: a model for virtual engagement in decentralized clinical trials. J Clin Transl Sci. 2023;7(1):e264,1–9. doi: 10.1017/cts.2023.671. PMC10789984 38229896

[7] Zawada SJ , Ruff KC , Sklar T , Demaerschalk BM. Towards a conceptual framework for addressing state-level barriers to decentralized clinical trials in the U.S. J Clin Transl Sci. 2023;7(1):e162. 37528942 10.1017/cts.2023.584 PMC10388410

[8] Dulko D , Kwong M , Palm M , Trinquart L , Selker H. From a decentralized clinical trial to decentralized and clinical-trial-in-a-box platform: towards patient-centric and equitable trials. J Clin Transl Sci. 2023;7(1):e236,1–9. doi: 10.1017/cts.2023.629. PMC10663768 38028335

[9] Cafaro T , LaRiccia PJ , Bandomer B , et al. Remote and semi-automated methods to conduct a decentralized randomized clinical trial. J Clin Transl Sci. 2023;7(1):e153. 37528946 10.1017/cts.2023.574 PMC10388435

[10] Avula N , Kakach D , Tignanelli CJ , et al. Strategies used for the COVID-OUT decentralized trial of outpatient treatment of SARS-CoV-2. J Clin Transl Sci. 2023;7(1):e242,1–7. doi: 10.1017/cts.2023.629. PMC10685265 38033705

[11] Soni A , Herbert C , Pretz C , et al. Design and implementation of a digital site-less clinical study of serial rapid antigen testing to identify asymptomatic SARS-CoV-2 infection. J Clin Transl Sci. 2023;7(1):e120. 37313378 10.1017/cts.2023.540 PMC10260333

[12] Cummins M , Soni H , Ivanova J , et al. Narrative review of telemedicine applications in decentralized research. J Clin Transl Sci. 2024; doi: 10.1017/cts.2024.3. PMC10880018 38384915

[13] Cummins M , Burr J , Young L , et al. Decentralized research technology use in U.S. multicenter clinical research studies based at academic research organizations. J Clin Transl Sci. 2023; e170,1–7. doi: 10.1017/cts.2023.678. PMC10790101 38229901

[14] United States Food and Drug Administration. (https://www.fda.gov/drugs/news-events-human-drugs/decentralized-clinical-trials-dct-draft-guidance-06202023). Accessed November 30, 2023.

